# Predictive Performance of Serum S100B *Versus* LDH in Melanoma Patients: A Systematic Review and Meta-Analysis

**DOI:** 10.3389/fonc.2021.772165

**Published:** 2021-12-07

**Authors:** Eszter Anna Janka, Tünde Várvölgyi, Zoltán Sipos, Alexandra Soós, Péter Hegyi, Szabolcs Kiss, Fanni Dembrovszky, Dezső Csupor, Patrik Kéringer, Dániel Pécsi, Margit Solymár, Gabriella Emri

**Affiliations:** ^1^ Department of Dermatology, Faculty of Medicine, University of Debrecen, Debrecen, Hungary; ^2^ Institute for Translational Medicine, Medical School, University of Pécs, Pécs, Hungary; ^3^ Doctoral School of Clinical Medicine, University of Szeged, Szeged, Hungary; ^4^ Department of Pharmacognosy, Faculty of Pharmacy, University of Szeged, Szeged, Hungary

**Keywords:** melanoma, S100B, LDH, ROC, COX, meta-analysis

## Abstract

**Background:**

Currently, no consensus on the use of blood tests for monitoring disease recurrence in patients with resected melanoma exists. The only meta-analysis conducted in 2008 found that elevated serum S100B levels were associated with significantly worse survival in melanoma patients. Serum LDH is an established prognostic factor in patients with advanced melanoma.

**Objective:**

To compare the discriminative and prognostic ability of serum S100B with that of serum LDH in patients with melanoma.

**Methods:**

This systematic review and meta-analysis were reported in accordance with the PRISMA Statement. The study protocol was registered in the International Prospective Register of Systematic Reviews (PROSPERO; CRD42019137138).

**Results:**

A quantitative analysis of data from 6 eligible studies included 1,033 patients with cutaneous melanoma. The discriminative ability of serum S100B at identifying disease relapse [pooled Area Under the ROC (AUROC) 78.64 (95% CI 70.28; 87.01)] was significantly greater than the discriminative ability of serum LDH [AUROC 64.41 (95% CI 56.05; 7278)] (p=0.013). Ten eligible studies with 1,987 patients were included in the risk of death analysis. The prognostic performance of serum S100B [pooled estimate of adjusted hazard ratio (HR) 1.78 (95% CI 1.38; 2.29)] was independent but not superior to that of serum LDH [HR 1.60 (95% CI 1.36; 2.29)].

**Limitations:**

A relatively small number of articles were eligible and there was considerable heterogeneity across the included studies.

**Conclusions:**

Serum biomarkers may provide relevant information on melanoma patient status and should be further researched. Serum S100B is a valid marker for diagnosis of melanoma recurrence.

**Systematic Review Registration:**

The study protocol was registered in the International Prospective Register of Systematic Reviews (PROSPERO; CRD42019137138).

## Introduction

The prevalence of melanoma is increasing worldwide among fair-skinned populations (1). Age-standardized world incidence rates are 0.33-35.8 per 100,000 according to the GLOBOCAN 2020 statistics (2). Melanoma is a cancer arising from the malignant transformation of pigment producing melanocytes. Ultraviolet radiation is an important risk factor for development of melanoma (3). However, the road from sun exposure to cutaneous melanoma is complex and diverse (3). Ultraviolet light is absorbed by nucleic acids, proteins or other endogenous chromophores, triggering biological processes in skin cells (3, 4). The degree of ultraviolet radiation induced stress and the protection against this stress are influenced both intracellular and intercellular molecular interactions (3). The interaction of variable environmental exposure and different genetic susceptibility and other host factors lead to the formation of melanomas with different biological behaviour and clinical characteristics (3, 5). In addition, melanoma derived proopiomelanocortin peptides, glucocorticoids, neurotransmitters, hormones, and intermediates of melanogenesis can affect the local and systemic immune responses, leading to tumor progression and therapy resistance (5). The synthesis of melanin is a tightly regulated multistep biochemical process (5). Melanogenesis can affect melanoma behaviour and disease outcome through regulation of cellular metabolism, and protecting melanoma cells against radiotherapy (6).

Melanoma is a tumor with a high risk of metastasis, and although disease relapse occurs most frequently in the first 3 years after resection of primary tumor, metastasis can occur any time and at any site (7). Thus, easily accessed (e.g., blood) cancer biomarkers for the early detection of disease relapse are urgently needed. The biomarkers should also provide prognostic information related to tumor biology and mirror tumor burden when traditional radiological criteria are not applicable to assess clinical benefit from therapy (8–10). Such biomarkers could improve patient outcomes. Furthermore, therapeutic response to immune checkpoint inhibitors or selective tyrosine kinase inhibitors is heterogeneous due to the complex interactions between the host and tumor (11–13). It is of great interest to identify biomarkers predicting clinical benefit from a particular therapy. Valid prognostic biomarkers associated with a specific aspect of tumor progression and metastasis are good candidates for such predictive models (11–14).

Serum lactate dehydrogenase (LDH) was the first prognostic blood biomarker to be included in the American Joint Committee on Cancer (AJCC) staging system for patients with metastatic melanoma3. In two meta-analyses performed by Petrelli et al. in 2015 and 2019, the prognostic effects of elevated serum LDH proved to be significant in melanoma (15, 16). Serum LDH correlates with tumor volume and necrosis and is not specific to tumor type (15). In addition, an elevation in serum LDH levels may correlate with tissue damage independent of malignancy. The tumor marker, S100B, is more specific to melanoma (9, 17, 18). Serum levels of S100B reflect tumor volume in metastatic disease; however, serum S100B levels can also be elevated in many other diseases, such as cardiovascular diseases, liver cirrhosis, migraine, chronic kidney disease, previous stroke, vitiligo, breast cancer, and SARS-CoV-2 infection (19–21). The only meta-analysis focused on serum S100B and melanoma showed that elevated serum S100B levels are associated with significantly worse survival in patients with melanoma (22).

Serum tumor markers usually have both prognostic and diagnostic predictive value to varying degrees (9, 10). From a diagnostic perspective, serum S100B levels are monitored in many cancer centers to detect disease relapse, while serum LDH is monitored less frequently in melanoma patients. A strong statistical correlation between S100B expression in melanoma tumor tissue samples and tumor stage has been found, and S100B protein is a possible target of therapeutic intervention (23–25). However, the estimates of sensitivity and specificity of serum S100B are highly variable (32-94% and 76-97%, respectively (26). Currently, no consensus on the use of blood tests for monitoring disease recurrence in patients with resected melanoma exists (27–30).

Although many serologic protein and non-protein markers that could aid early diagnosis of melanoma relapse as well as indicate patients’ prognosis have been reported, often primary studies are of variable quality and the findings are inconsistent (11, 23). Systematic reviews and meta-analyses are considered the reliable form to summarize the evidence about the prognostic and diagnostic value of particular factors (31). Meta-analysis to demonstrate whether serum S100B is a valid marker for the diagnosis of melanoma recurrence has not yet been published (23).

The objective of this study was to compare the prognostic and diagnostic abilities of serum S100B and serum LDH in patients with melanoma. Studies using Receiver Operating Characteristic (ROC) curves and Cox multivariate proportional-hazards models were included. The advantage of ROC is that the Area Under the ROC (AUROC) can be used to compare the accuracy of different diagnostic tests (32). The Cox regression model allows to detect and adjust for imbalance in prognostic variables; thus, it can be used to estimate more precisely a marker-dependent prognosis (33).

## Methods

This systematic review and meta-analysis were reported in accordance with the Preferred Reporting Items for Systematic Reviews and Meta-Analyses (PRISMA) Statement (31, 34, 35) (**Supplementary Table 1**: PRISMA-DTA Checklist). The study protocol was registered in the International Prospective Register of Systematic Reviews (PROSPERO; CRD42019137138).

### Deviation from the Registered Protocol

No subgroup analysis was planned. However, one eligible primary diagnostic effect study included patients with uveal melanoma, all other patients had cutaneous melanoma. Because the pathogenesis of uveal is different from cutaneous melanoma, the quantitative analysis was performed with the studies in which cutaneous melanoma patients were included. In addition, a diagnostic effect meta-analysis, which also included the study with uveal melanoma patients, was performed.

### Eligibility Criteria

Review questions were formulated using the PICOTS system according to the CHecklist for critical Appraisal and data extraction for systematic Reviews of prediction Modelling Studies (CHARMS) adapted to reviews of diagnostic effect studies and prognostic factor studies (31). The questions were formulated to determine whether elevated serum S100B is a more reliable marker than elevated serum LDH for predicting disease relapse in patients with different stages of melanoma and to determine whether elevated serum S100B is a more reliable marker than elevated serum LDH for predicting the risk of death and survival rates in metastatic melanoma. Articles providing information on S100B and LDH measurements at relapse confirmed by imaging and/or histopathological examination or overall risk of death and survival rates 1 and 2 years after S100B and LDH measurements were included. Studies assigning weights to the selected predictors (S100B and LDH) using Receiver Operating Characteristic (ROC) curves and Cox multivariate proportional-hazards models were included. The set of adjustment factors differed across primary prognostic studies. According to our pre-specifications, the studies included in the analysis used a minimum set of these factors: LDH and S100B plus at least one additional established prognostic marker, e.g., site of metastases or the presence of cerebral metastasis. If the patients enrolled in the study received therapy, we chose the results of the Cox model that was also adjusted for treatment. The findings should be useful for dermatologists and oncologists in the care of patients with melanoma.

### Search Strategy and Study Selection

MEDLINE, Embase, and the Cochrane Central Register of Controlled Trials were systematically searched from inception until January 15, 2021. The search included only English-language studies. Only the predictive factors in question and the targeted disease were used as keywords and terms for searching, including *S100B* or *S100* or *S-100B* or *S-100* and *lactate dehydrogenase* or *LDH* and *melanoma* in MEDLINE (*via* PubMed) and *melanoma* and *S100B* and *lactate dehydrogenase* in Embase and the Cochrane Central Register of Controlled Trials.

### Data Extraction

We followed the recommendations of CHARMS for data extraction (31). The items needed for the meta-analysis, assessment of applicability, and risk of bias were collected in Excel tables in a standard manner. First author and design of the study, the country where the study was conducted, and the year of publication, size of population (with and without metastasis, if applicable), inclusion and exclusion criteria for patient enrollment, demography (age, sex), information about the method and cut point of S100B and LDH measurement and reference test, the baseline prognostic factors used in Cox models, and outcome data of interest were extracted. Search, study selection, and data extraction were done by EAJ and GE, independently, and a consensus was reached through discussion.

### Assessment of Applicability and Risk of Bias (ROB)

Two authors (EAJ, GE) independently assessed study quality, and consensus was facilitated by flow diagrams for primary studies. To assess ROB and concerns regarding the applicability of diagnostic accuracy studies, the Quality Assessment of Diagnostic Accuracy Studies (QUADAS-2) tool was used (36). ROB of prognostic factor studies was assessed according to the Quality In Prognosis Studies (QUIPS) tool (37).

### Statistical Analysis

Heterogeneity across studies was assessed using the I² statistics, where I² = 100% × (Q − df)/Q and represents the magnitude of the heterogeneity (moderate: 30–60%; substantial: 50–90%; considerable: 75–100%) (38). Pooled estimates (AUROC with 95% confidence intervals, sensitivity, specificity, adjusted HR with 95% confidence intervals, survival rates (1-year, 2-year) with 95% confidence intervals) were calculated using a DerSimonian-Laird random-effect model (39). Funnel plots and Egger’s tests were applied to access the presence of publication bias. Statistical analyses were performed with Stata 16.1 data analysis and statistical software (Stata Corp LLC, College Station, TX, USA) and R package, version 4.0.3. (R Foundation for Statistical Computing).

## Results

### Study Selection and Characteristics of Included Studies

**Figure 1:**
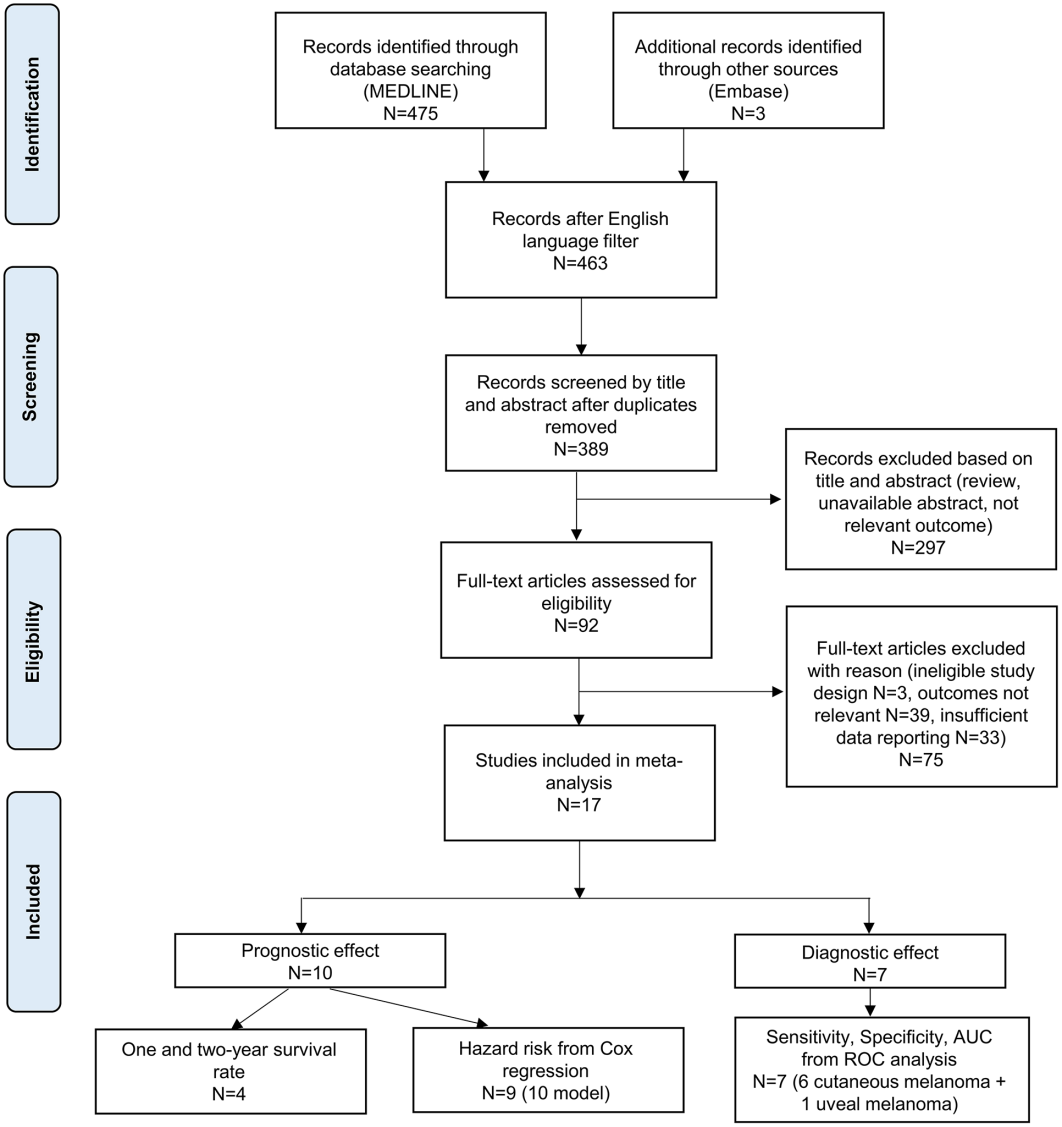
PRISMA flowchart. AUC, area under curve; ROC, Receiver Operating Characteristic.

**Figure 2:**
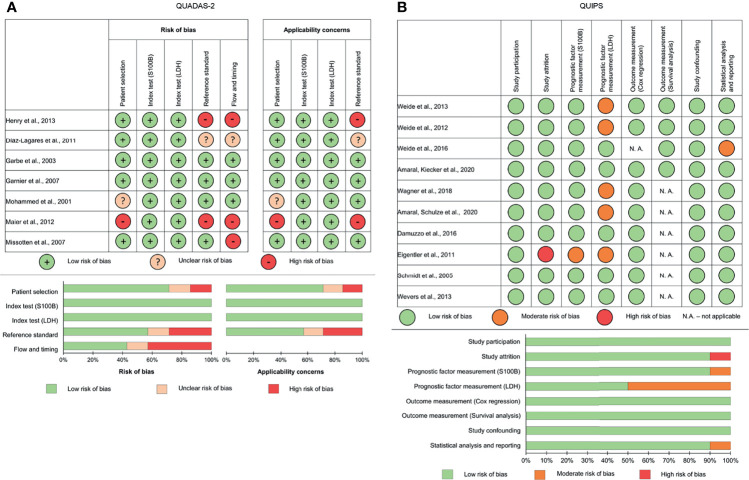
Results of quality assessment according to the Quality Assessment of Diagnostic Accuracy Studies (QUADAS-2) **(A)** and Quality In Prognosis Studies (QUIPS) **(B)** tools.

The literature search yielded 478 records (**Figure 1**). After the removal of non-English-language studies and duplicates, 389 articles remained. Based on titles or abstracts, 92 articles were selected for full-text screening. Thirteen full-texts were not available and 62 did not meet eligibility criteria. Finally, 7 primary diagnostic effect studies (6 cutaneous melanoma, 1 uveal melanoma) (40–46) and 10 primary prognostic factor studies (47–56) were selected for the qualitative and quantitative synthesis. Characteristics of the included studies are summarized in **Tables 1**–**3** and **Figure 2**.

**Table 1:** Characteristics of included diagnostic effect studies in the meta-analysis.

AUC (ROC analysis), Sensitivity, Specificity												
First author (Year of publication)	Country	Design of the study	Settings	Population	Female %	S100B cutoff (µg/L)	S100B methods	LDH cutoff (IU/L)	LDH methods	Total number of patients	No. of patients with regional or distant metastasis	No. of patients without regional or distant metastasis
Henry et al., 2012 (40)	France	prospective	single center	Stage I-IV melanoma (unknown SLN status, stage I-II at inclusion 44%)	41.3	0.15	LIAISON^®^ Sangtec^®^ 100	ULN (240)	automated colourimetric assay	121	43	78
Díaz-Lagares et al., 2011 (41)	Spain	retrospective	single center	Stage I-IV melanoma	54	0.1	Elecsys^®^ S100	ULN (292)	automated colourimetric assay	176	110	66
Garbe et al., 2003 (42)	Germany	prospective	single center	Stage II-III melanoma (unknown SLN status, stage II at inclusion 56%)	56.8	0.12	LIA-mat^®^ Sangtec^®^ 100	ULN (240)	automated colourimetric assay	296	41	255
Garnier et al., 2007 (43)	France	prospective	single center	Stage I-IV melanoma (stage I-II at inclusion 34%)	46.5	0.12	LIA-mat^®^ Sangtec^®^ 100	ULN (439)	automated colourimetric assay	170	113	57
Mohammed et al., 2001 (44)	United Kingdom	prospective	single center	Stage I-IV melanoma (stage I-II at inclusion 12%)	50.6	0.15	LIA-mat^®^ Sangtec^®^ 100	ULN (500)	automated colourimetric assay	164	85	79
Maier et al., 2012 (45)	Germany	retrospective	single center	Stage I-IV melanoma	43.4	0.11	Elecsys^®^ S100	ULN (250)	automated colourimetric assay	106	24	82
Missotten et al., 2007 (46)	The Netherlands	retrospective	single center	Nonmetastatic and metastatic uveal melanoma	N.R.	0.16	LIAISON^®^ Sangtec^®^ 100	ULN (450)	automated colourimetric assay	134	30	104

SLN, sentinel lymph node; ULN, upper limit normal; N.R., not reported.

Cutoff levels for serum S100B were selected as the 95th percentile of the control group defined by the manufacturer (40, 43, 44) or a previous report (45), or determined by including healthy individuals in the study (41, 42, 46). ROC optimized cutoffs were reported in only a few studies (40, 43). Colorimetric assays were used in all selected studies for determining serum LDH. The cutoff was usually the upper limit of the normal (ULN) level as defined by the local laboratory.

**Table 2:** Characteristics of included prognostic effect (Cox regression) studies in the meta-analysis.

Hazard risk (Cox regression)										
First author (Year of publication)	Country	Design of the study	Settings	Population	Female %	S100B cutoff (µg/L)	S100B methods	LDH cutoff (IU/L)	LDH methods	Total number of patients
Weide et al., 2012 (47)	Germany	prospective	multicenter	Resectable and nonresectable stage IV	43.6	0.15; 0.10	Sangtec^®^ 100 ELISA, Elecsys^®^ S100	ULN	automated colourimetric assay	586
Weide et al., 2013 (48)	Germany	prospective	multicenter	Nonresectable stage IV with first-line systemic therapy	41.5	0.15; 0.10	Sangtec^®^ 100 ELISA, Elecsys^®^ S100	ULN	automated colourimetric assay	372
Wagner cohort 1, 2018 (51)	Germany	retrospective	single center	Nonresectable stage III/stage IV with anti-PD1 therapy	42.1	0.3	N.R.	1.5xULN	automated colourimetric assay	152
Wagner cohort 2, 2018 (51)	Germany	retrospective	single center	Nonresectable stage III/stage IV with anti-PD1 + anti-CTLA4 therapy	41.9	0.3	N.R.	1.5xULN	automated colourimetric assay	86
Amaral, Kiecker et. al., 2020 (50)	Germany	retrospective	multicenter	Nonresectable stage IV (brain met) with combined immunotherapy	36.8	0.11	Elecsys^®^ S100	250	automated colourimetric assay	265/322
Amaral, Schulze et. al., 2020 (52)	Germany	prospective	single center	Nonresectable stage IV with combined immunotherapy	39	0.15	LIA-mat^®^ Sangtec^®^ 100	ULN	automated colourimetric assay	55/59
Damuzzo et al., 2016 (53)	Italy	prospective	single center	Nonresectable stage IV with anti-CTLA-4 therapy	34.1	0.16	LIAISON^®^ Sangtec^®^ 100	450	automated colourimetric assay	44
Eigentler et al., 2011 (54)	Germany	retrospective	multicenter	Nonresectable stage IV (brain metastasis)	44	ULN	N.R.	ULN	automated colourimetric assay	270/464
Wevers et al., 2013 (55)	The Netherlands	prospective	single center	Resectable stage III	47.1	0.15, 0.20	Nichols Advantage, Sangtec^®^ 100 ELISA	250	automated colourimetric assay	75
Schmidt et al., 2005 (56)	Denmark	retrospective	single center	Nonresectable stage IV treated with IL2-based immunotherapy	44	0.15	LIAISON^®^ Sangtec^®^ 100	500	automated colourimetric assay	82

SLN, sentinel lymph node; ULN, upper limit normal; N.R., not reported.

**Table 3:** Characteristics of included prognostic effect (Survival rate) studies in the meta-analysis.

Survival rate (one- and two-year)										
First author (Year of publication)	Country	Design of the study	Settings	Population	Female %	S100B cutoff (µg/L)	S100B methods	LDH cutoff (IU/L)	LDH methods	Total number of patients
Weide et al., 2012 (47)	Germany	prospective	multicenter	Resectable and nonresectable stage IV	43.6	0.15; 0.10	Sangtec^®^ 100 ELISA, Elecsys^®^ S100	ULN	automated colourimetric assay	855
Weide et al., 2013 (48)	Germany	prospective	multicenter	Nonresectable stage IV with first-line systemic therapy	41.5	0.15; 0.10	Sangtec^®^ 100 ELISA, Elecsys^®^ S100	ULN	automated colourimetric assay	499
Weide et al., 2016 (49)	Germany	prospective	multicenter	Nonresectable stage IV	41.3	0.10	Elecsys^®^ S100	250	automated colourimetric assay	206
Amaral, Kiecker et. al., 2020 (50)	Germany	retrospective	multicenter	Nonresectable stage IV (brain metastasis) with combined immunotherapy	36.8	0.11	Elecsys^®^ S100	250	automated colourimetric assay	380

SLN, sentinel lymph node; ULN, upper limit normal; N.R., not reported.

### Quality of the Included Studies

The qualitative evaluation demonstrated that many studies were performed with bias; the greatest risk of bias was found in the study reference standards. Imaging techniques with different sensitivities and specificities as a reference standard for detection of disease relapse varied depending on the stage in the diagnostic accuracy studies. Because not all domains could be rated as having low ROB, the overall judgment was avoided. Publication bias was unlikely according to the Funnel plot for AUROC (**Supplementary Figures 1**, **2**). The Funnel plot and Egger’s test did not verify publication bias for Cox hazard ratios (p=0.245 for S100B; p=0.344 for LDH) (**Supplementary Figure 3**).

### Diagnostic Effect Meta-Analysis

**Figure 3:**
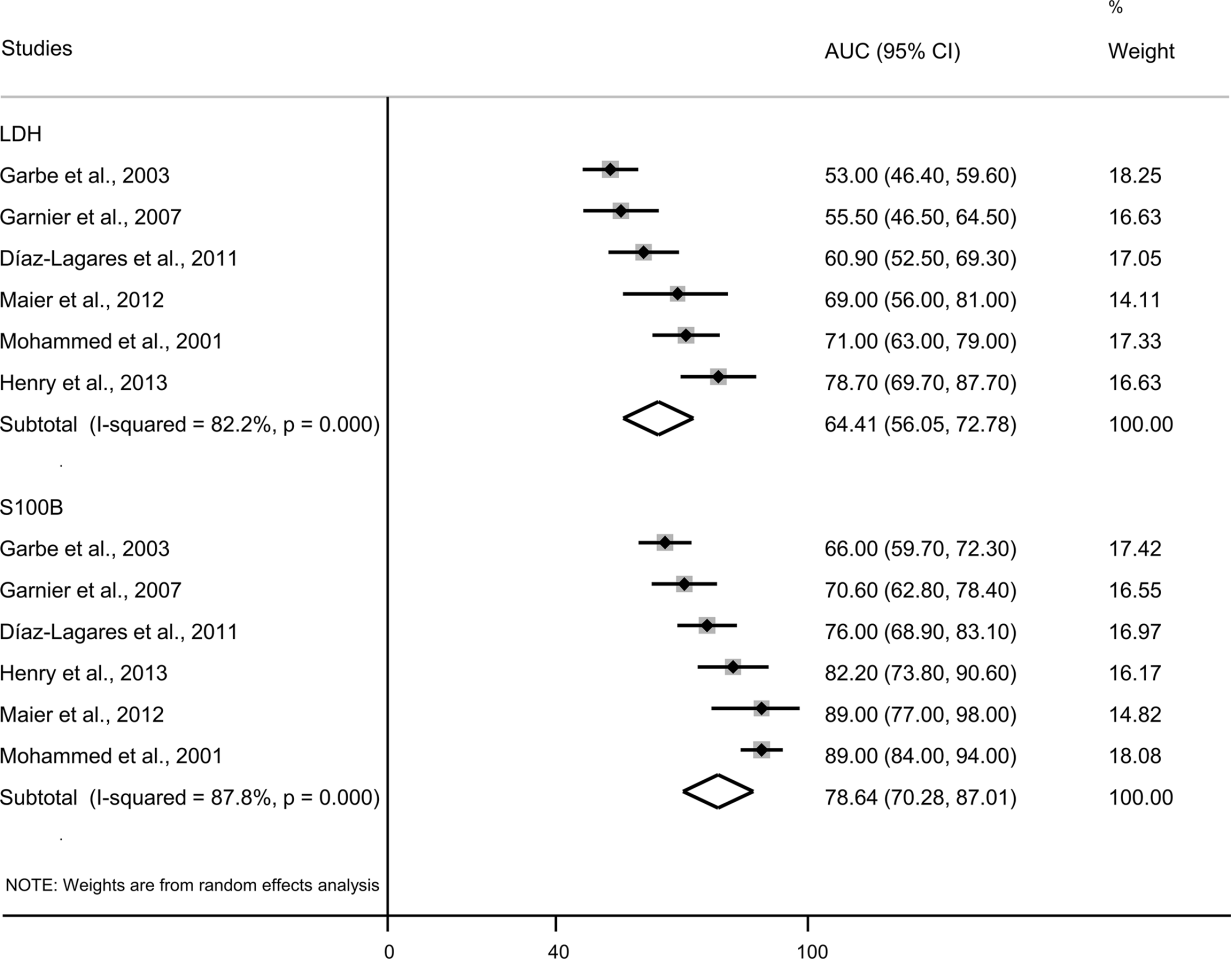
Forest plot presenting AUC with 95% CI from ROC curve for S100B and LDH. AUC, area under the curve; CI, confidence intervals; LDH, lactate dehydrogenase.

Six eligible studies with 1,033 patients with cutaneous melanoma were included in the meta-analysis. The quantitative evaluation showed that discriminative ability of S100B to correctly identify patients with or without melanoma relapse [AUROC 78.64 (70.28; 87.01)] was significantly (p=0.013) greater than the discriminative ability of LDH [AUROC 64.41 (56.05; 72.78)] (**Figure 3**). In addition, sensitivity and specificity were analyzed in these studies using predefined cut-off points (**Table 1**) for the dichotomized continuous values of serum S100B and LDH. The pooled sensitivity of S100B [61.35% (95% CI 48.90; 73.80)] was significantly higher (p=0.017) than the pooled sensitivity of LDH [33.93% (95% CI 17.21; 50.65)] (**Supplementary Figure 4**). The pooled specificity of S100B [87.30% (95% CI 81.10; 93.49)] was similar (p=0.557) to the pooled specificity of LDH [90.70% (95% CI 84.89; 96.51] (**Supplementary Figure 5**). The ROC optimized cut-off point for serum S100B was higher than the cutoff predefined by the manufacturer and was associated with higher specificity, but lower sensitivity (40, 43).

A quantitative analysis of data from 7 eligible studies included 1,167 participants (n=1,033 cutaneous melanoma, n=134 uveal melanoma). The discriminative ability of serum S100B to correctly identify patients with or without disease relapse [AUROC 79.75 (95% CI 72.28; 87.21)] did not differ significantly (p=0.061) from the discriminative ability of serum LDH [AUROC 68.18 (95% CI 57.65; 78.69)] (**Supplementary Figure 6**). The pooled sensitivity of serum S100B [61.37% (95% CI 50.21; 72.54)] was significantly higher (p=0.024) than the sensitivity of LDH [37.47% (95% CI 21.20; 53.73)] (**Supplementary Figure 7**). The pooled specificity of S100B [89.22% (95% CI 84.00; 94.43)] was similar (p=0.643) to the pooled specificity of LDH [91.25% (95% CI 86.40; 96.10)] (**Supplementary Figure 8**).

### Prognostic Effect Meta-Analysis

**Figure 4:**
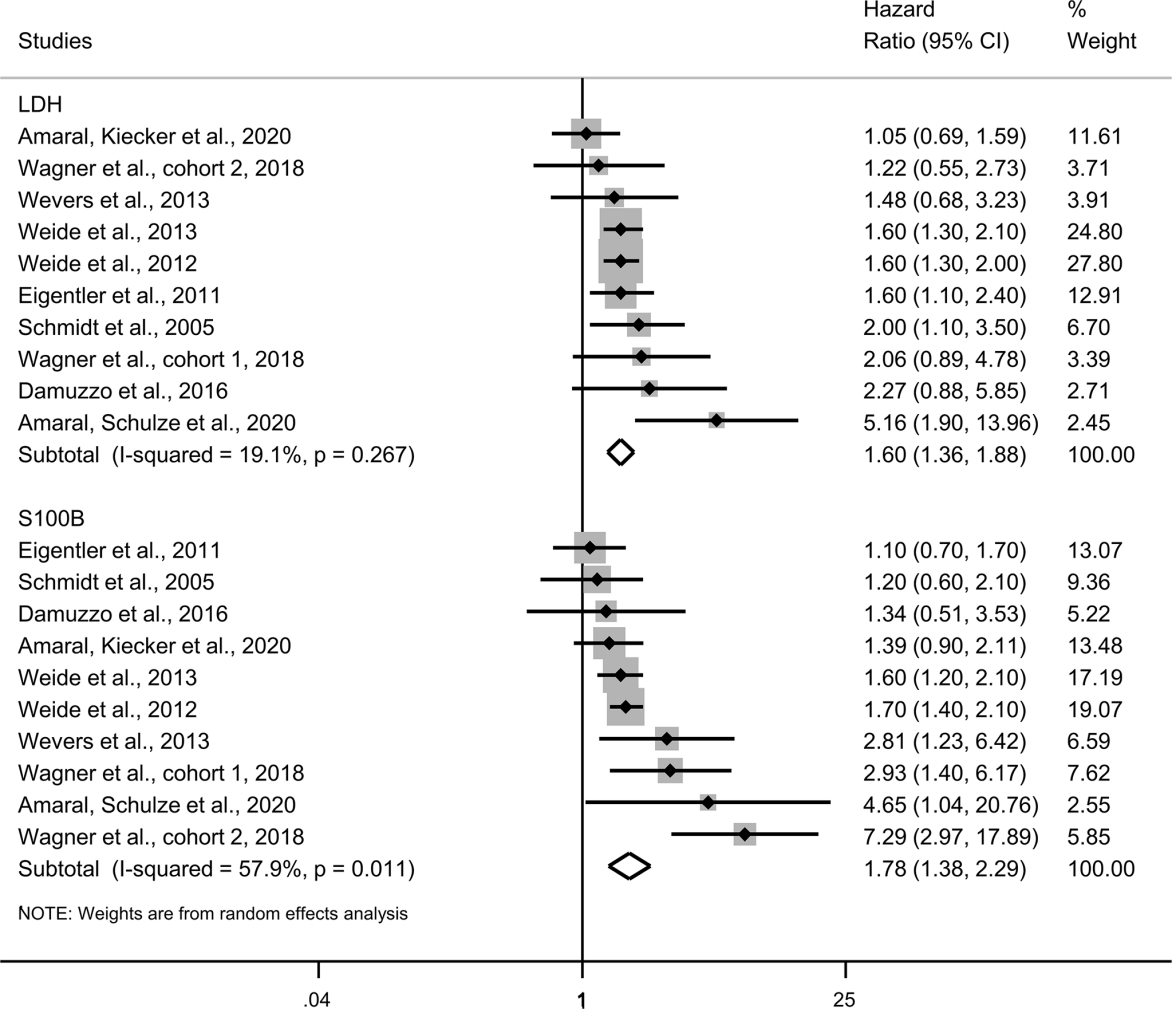
Forest plot presenting adjusted hazard ratios with 95% CI from Cox multivariate proportional-hazards models of overall survival. CI, confidence intervals; LDH, lactate dehydrogenase.

Ten eligible studies with 1,987 participants were included (**Table 2**) in the adjusted hazard ratios analysis using the Cox multivariate proportional-hazards models of overall survival (**Figure 4**). There were no significant differences between the hazard ratios associated with elevated serum S100B levels [1.78 (1.38; 2.29)] and the hazard ratios of elevated LDH levels [1.60 (1.36; 1.88)] (p=0.389). Both elevated serum S100B levels and elevated LDH levels predicted a higher risk of death in patients with metastatic melanoma.

Four eligible studies with 1,940 participants were included in the analysis of one-, and two-year survival rates (**Table 3**). The pooled one-year survival rate of patients with normal serum S100B levels was significantly higher [55.92% (39.91%; 71.92%)] than the one-year survival rate of patients with elevated serum S100B levels [28.08% (10.83%; 45.34%)] (p=0.033) (**Supplementary Figure 9**). A similar trend was observed for the two-year survival rate [normal serum S100B: 32.51% (24.36%; 40.67%); elevated serum S100B: 14.68% (5.77%; 23.58%)], but the difference was not significant (p=0.082) (**Supplementary Figure 10**). The one-year survival rate was higher for patients with normal serum LDH levels [46.16% (29.25%; 63.06%)] than the one-year survival rate for patients with elevated serum LDH levels [25.94 (8.15%; 43.72%)], but the difference was not significant (p=0.152) (**Supplementary Figure 11**). The results for the two-year survival rate were similar [normal LDH levels: 26.94% (17.96%; 35.93%); elevated LDH levels: 13.39% (5.04%; 21.74)] (p=0.207) (**Supplementary Figure 12**). We found no significant differences between the prognostic performance of serum S100B and serum LDH for predicting one-year (p=0.886) (**Supplementary Figure 13**) or two-year (p=0.921) survival rates (**Supplementary Figure 14**).

## Discussion

Intracellular S100 proteins are Ca2+- and Zn2+-sensors involved in several protein interactions regulating a wide variety of cellular processes, including transcription, protein phosphorylation, motility, energy metabolism, which may affect tumor growth (57–59). In addition, extracellular S100B is a damage-associated molecular pattern protein, which may promote tumor progression by contributing to cancer-associated inflammation or by activating signaling pathways in melanoma cells *via* receptors for advanced glycation end products (57–59). The main source of elevated serum S100B levels in melanoma is the passive release from damaged/necrotic cells; however, the same tumor burden may or may not cause an elevation of S100B serum levels (57). In tumor cells dependent on glycolysis, lactate production increases substantially due to the increased expression and activity of LDH, which converts pyruvate to lactate. Lactate, which is exported by tumor cells, may promote angiogenesis, metastasis, therapy resistance, and immunosuppression (60). In malignant cells at the more oxygenated tumor periphery, lactate is utilized as an energetic source; lactate must be converted to pyruvate *via* LDH for this purpose (60, 61). Elevated serum LDH in patients with advanced melanoma is primarily due to release from glycolytic tumor cells (LDH3 and 4) (61).

In our meta-analysis, the pooled AUROC for correctly identifying disease relapse proved to be significantly higher for serum S100B than for serum LDH, indicating that serum S100B is a more suitable marker for tumor recurrence during follow-up of patients with cutaneous melanoma. Of note, S100B is the only serum biomarker supported by sufficient data that is routinely available in most hospitals. The serum S100B concentration was shown to be significantly higher in patients of stage III or IV than in those of stages I and II, and significantly higher in patients of stage IV than stage III (62). Serum S100B, however, seemed incapable of predicting sentinel lymph node status (63). Importantly, Abraha et al. found that diagnostic accuracy for detecting advanced disease may be higher by combining an elevated serum S100B and a Breslow tumor thickness of >4mm (62). Elevated levels of serum S100B occur in a number of conditions (19); thus, these findings support that monitoring S100B is recommended primarily in cases of melanoma with a high risk of relapse. Nevertheless, when a pre-specified cutoff (the upper limit of normal or ROC optimized) was used, serum S100B and LDH proved to be similarly and highly specific and moderately sensitive; however, the sensitivity of serum S100B was significantly higher compared to serum LDH. This result suggests that monitoring serum S100B might indicate the need for an imaging examination to detect disease relapse earlier than serum LDH. Further studies, both in clinical trials and in real-world populations, are needed to clarify how the measurement method, cut point, reference test, and patient population affect the accuracy of serum S100B for the detection of disease recurrence. These studies could also explore the sources of the considerable heterogeneity observed in our meta-analysis (31). Nevertheless, melanoma is heterogeneous in terms of biological behavior, due to the heterogeneity of the genome and proteome; the identification of a single biomarker that can be used widely is difficult (3). Further studies are needed to identify additional biomarkers that could be used in combination with serum S100B to increase the chances of early detection of disease relapse (45).

A number of circulating biomarkers are being investigated that may help us in follow-up. Compared to tissue tumor biopsy, peripheral blood sample (liquid biopsy) is more readily available and less heterogeneous (13). Many serologic markers such as enzymes [e.g., matrix metalloproteinase-9 (MMP-9)], secreted proteins (e.g., melanoma inhibiting activity), metabolites of the melanin synthesis pathway (e.g., 5-S-cysteinyl-dopa), circulating nucleic acids (e.g., tyrosinase mRNA, circulating-free DNA BRAFV600E mutation), and peripheral blood immune markers (e.g., soluble PD-L1) have been shown to correlate with tumor progression, survival or response to treatment in patients with melanoma (9–14, 23, 30, 64, 65). Properly designed, conducted, analyzed and reported prediction model studies will determine how to use these markers with the greatest clinical benefit (11, 31, 66, 67).

In a subgroup of patients with metastatic melanoma, the levels of serum S100B were not elevated and many studies and reviews have been published on the prognostic effect of serum S100B (17, 18, 29, 68–70). Because of the strong discriminative ability of serum S100B in identifying metastatic disease, the inclusion of studies on patients with all stages of melanoma was considered to be inappropriate for prognostic effect analysis; thus, only studies in which metastatic patients were included were selected. Surprisingly, very few eligible studies were identified because of the scarcity of multivariate analyses, patient selection bias, and significant reporting bias on outcomes in prognostic studies in the field. The Cox regression models that were included in the analysis used established prognostic markers as adjustment factors, e.g., site of metastases, the presence of brain metastasis, treatment. According to our results, the summary adjusted hazard ratio for S100B was similar to that for LDH, i.e. serum S100B has a similar prognostic value as serum LDH in patients with metastatic melanoma. Importantly, in accordance with the different biology coupled with elevated levels of serum LDH and S100B, the studies included in the meta-analysis indicated that the prognostic ability of the two markers was independent. Reviewing the literature, we found only one meta-analysis that examined the association between serum S100B levels and melanoma prognosis. In this meta-analysis, elevated serum S100B levels were associated with significantly poorer survival in melanoma patients (22). MMP-9 plays an important role in melanoma invasiveness. In one study, elevated serum MMP-9 levels and the circulating-free DNA BRAFV600E mutation were found to be associated with poor progression-free survival and overall survival. MMP-9 may be a promising indicator of the response to BRAF inhibitors in combination with the detection of the BRAFV600E mutation (12). The programmed cell death protein 1/programmed cell death ligand 1 (PD-1/PD-L1) axis plays an important role in circumventing immune surveillance. There is a need for a biomarker that would predict the efficacy of PD-1/PD-L1 inhibitors in patients with metastatic melanoma. Yue C et al. found that a decrease in circulating PD-L1 + tumor cell count was associated with a strong antitumor response. Also, patients with high levels of PD-L1 + circulating tumor cells at baseline are generally susceptible to anti-PD-L1 therapy (71). Since serum S100B and LDH monitoring also appear to be prognostically useful in melanoma patients during BRAF-inhibitor or immune checkpoint inhibitor treatment (69, 70, 72, 73), the combination of these markers could be further evaluated in predictive models identifying subgroups with differential treatment effects. The novelty of this meta-analysis was the comparative approach, the analysis of multiple outcomes, and the inclusion of logistic regression models. Furthermore, the results were derived from the analysis of data from patient populations with more than 1,000 participants for each of the studied outcomes.

## Limitations

A high risk of bias regarding statistical analysis and reporting domain was detected in many predictive studies screened for analysis, but the bias was lower in the selected studies due to the applied inclusion criteria. This, in turn, led to only a few articles being eligible for data extraction, which is a limitation of this meta-analysis. In addition, there was considerable heterogeneity across the included studies. The immunoassays used for measuring serum S100B and the cutoff for determining normal *versus* elevated S100B levels were not completely uniform across studies. The adjustment factors in the prognostic studies were also not uniform. A limitation of this review is that a majority of eligible prognostic studies came from Germany (German Central Malignant Melanoma Registry), although the data were collected from different periods and/or from an intentionally chosen different setting. Our attempt to contact the first author to obtain information on the extent of potential overlap between populations of prognostic factor studies performed by the same research group was unsuccessful.

## Conclusions

The applicability of serum S100B and serum LDH for predicting the progression of melanoma was studied in this review from both diagnostic and prognostic viewpoints. We found that the discriminative ability of serum S100B at identifying disease relapse was greater than that of serum LDH. Since a relapse of melanoma is associated with elevated serum S100B levels in only a subset of patients, serum S100B should be considered in combination with additional serum biomarkers in a multivariable diagnostic prediction model. Furthermore, serum S100B had a similar and independent prognostic strength in metastatic melanoma compared with serum LDH, suggesting that the implementation of both markers in a multivariable prognostic prediction model development would be advantageous. To increase the degree of confidence in the prognostic and diagnostic abilities of various biomarkers, primary predictor studies conducted and reported in accordance with the corresponding quality assessment tools are important.

## Data Availability Statement

The original contributions presented in the study are included in the article/**Supplementary Material**. Further inquiries can be directed to the corresponding author.

## Author Contributions

Concept and design: EJ, PH, ZS, and GE. Review questions were formulated by EJ and GE, guided by PH and DP. The applicability of the selected studies to the review question was determined by two authors (EJ and GE), independently, then a consensus was reached through discussion. Search, study selection, and data extraction: EJ and GE. Risk of bias assessment: EJ and GE. Statistical analysis: ZS and AS. Drafting of the manuscript: EJ, TV, and GE. Critical revision of the manuscript: all authors. All authors contributed to the article and approved the submitted version.

## Funding

This work was supported by the ÚNKP-20-4 New National Excellence Program of the Ministry for Innovation and Technology from the source of the National Research, Development and Innovation Fund. Furthermore, the project was supported by the European Union and the European Regional Development Fund GINOP-2.3.2-15-2016-00005 and Hungarian Scientific Research Fund NKFIH K120206.

## Conflict of Interest

The authors declare that the research was conducted in the absence of any commercial or financial relationships that could be construed as a potential conflict of interest.

## Publisher’s Note

All claims expressed in this article are solely those of the authors and do not necessarily represent those of their affiliated organizations, or those of the publisher, the editors and the reviewers. Any product that may be evaluated in this article, or claim that may be made by its manufacturer, is not guaranteed or endorsed by the publisher.

## References

[1] Schadendorf D van Akkooi ACJ Berking C Griewank KG Gutzmer R Hauschild A . Melanoma. Lancet (London England) (2018) 392(10151):971–84. doi: 10.1016/S0140-6736(18)31559-9 30238891

[2] IARC . International Agency for Research on Cancer, 2020. Lyon (2020). Available at: http://www.iarc.fr/ (Accessed Last 14 May 2021).

[3] Emri G Paragh G Tosaki A Janka E Kollar S Hegedus C . Ultraviolet Radiation-Mediated Development of Cutaneous Melanoma: An Update. J Photochem Photobiol B (2018) 185:169–75. doi: 10.1016/j.jphotobiol.2018.06.005 29936410

[4] Slominski AT Zmijewski MA Plonka PM Szaflarski JP Paus R . How UV Light Touches the Brain and Endocrine System Through Skin, and Why. Endocrinology (2018) 159(5):1992–2007. doi: 10.1210/en.2017-03230 29546369 PMC5905393

[5] Slominski AT Carlson JA . Melanoma Resistance: A Bright Future for Academicians and a Challenge for Patient Advocates. Mayo Clin Proc (2014) 89(4):429–33. doi: 10.1016/j.mayocp.2014.02.009 PMC4050658 24684870

[6] Slominski RM Zmijewski MA Slominski AT . The Role of Melanin Pigment in Melanoma. Exp Dermatol (2015) 24(4):258–9. doi: 10.1111/exd.12618 PMC4450257 25496715

[7] Gershenwald JE Scolyer RA Hess KR Sondak VK Long GV Ross MI . Melanoma Staging: Evidence-Based Changes in the American Joint Committee on Cancer Eighth Edition Cancer Staging Manual. CA Cancer J Clin (2017) 67(6):472–92. doi: 10.3322/caac.21409 PMC5978683 29028110

[8] Aide N Hicks RJ Le Tourneau C Lheureux S Fanti S Lopci E . FDG PET/CT for Assessing Tumour Response to Immunotherapy : Report on the EANM Symposium on Immune Modulation and Recent Review of the Literature. Eur J Nucl Med Mol Imaging (2019) 46(1):238–50. doi: 10.1007/s00259-018-4171-4 PMC6267687 30291373

[9] Vereecken P Cornelis F Van Baren N Vandersleyen V Baurain JF . A Synopsis of Serum Biomarkers in Cutaneous Melanoma Patients. Dermatol Res Pract (2012) 2012:260643. doi: 10.1155/2012/260643 22287956 PMC3263591

[10] Wakamatsu K Fukushima S Minagawa A Omodaka T Hida T Hatta N . Significance of 5-S-Cysteinyldopa as a Marker for Melanoma. Int J Mol Sci (2020) 21(2):432. doi: 10.3390/ijms21020432 PMC7013534 31936623

[11] Kitano S Nakayama T Yamashita M . Biomarkers for Immune Checkpoint Inhibitors in Melanoma. Front Oncol (2018) 8:270. doi: 10.3389/fonc.2018.00270 30073150 PMC6058029

[12] Salemi R Falzone L Madonna G Polesel J Cina D Mallardo D . MMP-9 as a Candidate Marker of Response to BRAF Inhibitors in Melanoma Patients With BRAF(V600E) Mutation Detected in Circulating-Free DNA. Front Pharmacol (2018) 9:856. doi: 10.3389/fphar.2018.00856 30154717 PMC6102751

[13] Yi M Jiao D Xu H Liu Q Zhao W Han X . Biomarkers for Predicting Efficacy of PD-1/PD-L1 Inhibitors. Mol Cancer (2018) 17(1):129. doi: 10.1186/s12943-018-0864-3 30139382 PMC6107958

[14] Zhou J Mahoney KM Giobbie-Hurder A Zhao F Lee S Liao X . Soluble PD-L1 as a Biomarker in Malignant Melanoma Treated With Checkpoint Blockade. Cancer Immunol Res (2017) 5(6):480–92. doi: 10.1158/2326-6066.CIR-16-0329 PMC5642913 28522460

[15] Petrelli F Cabiddu M Coinu A Borgonovo K Ghilardi M Lonati V . Prognostic Role of Lactate Dehydrogenase in Solid Tumors: A Systematic Review and Meta-Analysis of 76 Studies. Acta Oncol (2015) 54(7):961–70. doi: 10.3109/0284186X.2015.1043026 25984930

[16] Petrelli F Ardito R Merelli B Lonati V Cabiddu M Seghezzi S . Prognostic and Predictive Role of Elevated Lactate Dehydrogenase in Patients With Melanoma Treated With Immunotherapy and BRAF Inhibitors: A Systematic Review and Meta-Analysis. Melanoma Res (2019) 29(1):1–12. doi: 10.1097/CMR.0000000000000520 30308577

[17] Kruijff S Hoekstra HJ . The Current Status of S-100B as a Biomarker in Melanoma. Eur J Surg Oncol (2012) 38(4):281–5. doi: 10.1016/j.ejso.2011.12.005 22240030

[18] Harpio R Einarsson R . S100 Proteins as Cancer Biomarkers With Focus on S100B in Malignant Melanoma. Clin Biochem (2004) 37(7):512–8. doi: 10.1016/j.clinbiochem.2004.05.012 15234232

[19] Heizmann CW . S100 Proteins: Diagnostic and Prognostic Biomarkers in Laboratory Medicine. Biochim Biophys Acta Mol Cell Res (2019) 1866(7):1197–206. doi: 10.1016/j.bbamcr.2018.10.015 30392897

[20] Gebhardt C Lichtenberger R Utikal J . Biomarker Value and Pitfalls of Serum S100B in the Follow-Up of High-Risk Melanoma Patients. J Dtsch Dermatol Ges (2016) 14(2):158–64. doi: 10.1111/ddg.12727 26819111

[21] Aceti A Margarucci LM Scaramucci E Orsini M Salerno G Di Sante G . Serum S100B Protein as a Marker of Severity in Covid-19 Patients. Sci Rep (2020) 10(1):18665. doi: 10.1038/s41598-020-75618-0 33122776 PMC7596559

[22] Mocellin S Zavagno G Nitti D . The Prognostic Value of Serum S100B in Patients With Cutaneous Melanoma: A Meta-Analysis. Int J Cancer (2008) 123(10):2370–6. doi: 10.1002/ijc.23794 18752249

[23] Tandler N Mosch B Pietzsch J . Protein and Non-Protein Biomarkers in Melanoma: A Critical Update. Amino Acids (2012) 43(6):2203–30. doi: 10.1007/s00726-012-1409-5 23053020

[24] Leclerc E Heizmann CW Vetter SW . RAGE and S100 Protein Transcription Levels Are Highly Variable in Human Melanoma Tumors and Cells. Gen Physiol Biophys (2009) 28 Spec No Focus:F65–75. 20093728

[25] Wu KJ Ho SH Wu C Wang HD Ma DL Leung CH . Simultaneous Blocking of the Pan-RAF and S100B Pathways as a Synergistic Therapeutic Strategy Against Malignant Melanoma. J Cell Mol Med (2021) 25(4):1972–81. doi: 10.1111/jcmm.15994 PMC7882986 33377602

[26] Ertekin SS Podlipnik S Ribero S Molina R Rios J Carrera C . Monthly Changes in Serum Levels of S100B Protein as a Predictor of Metastasis Development in High-Risk Melanoma Patients. J Eur Acad Dermatol Venereology JEADV (2020) 34(7):1482–8. doi: 10.1111/jdv.16212 31967695

[27] Dummer R Hauschild A Lindenblatt N Pentheroudakis G Keilholz U Committee EG . Cutaneous Melanoma: ESMO Clinical Practice Guidelines for Diagnosis, Treatment and Follow-Up. Ann Oncol (2015) 26 Suppl 5:v126–32. doi: 10.1093/annonc/mdv297 26314774

[28] Coit DG Thompson JA Albertini MR Barker C Carson WE Contreras C . Cutaneous Melanoma, Version 2.2019, NCCN Clinical Practice Guidelines in Oncology. J Natl Compr Canc Netw (2019) 17(4):367–402. doi: 10.6004/jnccn.2019.0018 30959471

[29] Beyeler M Waldispuhl S Strobel K Joller-Jemelka HI Burg G Dummer R . Detection of Melanoma Relapse: First Comparative Analysis on Imaging Techniques *Versus* S100 Protein. Dermatol (Basel Switzerland) (2006) 213(3):187–91. doi: 10.1159/000095034 17033166

[30] Revythis A Shah S Kutka M Moschetta M Ozturk MA Pappas-Gogos G . Unraveling the Wide Spectrum of Melanoma Biomarkers. Diagnostics (Basel) (2021) 11(8):1341. doi: 10.3390/diagnostics11081341 34441278 PMC8391989

[31] Riley RD Moons KGM Snell KIE Ensor J Hooft L Altman DG . A Guide to Systematic Review and Meta-Analysis of Prognostic Factor Studies. BMJ (Clinical Res ed) (2019) 364:k4597. doi: 10.1136/bmj.k4597 30700442

[32] Jones CM Athanasiou T . Summary Receiver Operating Characteristic Curve Analysis Techniques in the Evaluation of Diagnostic Tests. Ann Thorac Surg (2005) 79(1):16–20. doi: 10.1016/j.athoracsur.2004.09.040 15620907

[33] Christensen E . Multivariate Survival Analysis Using Cox's Regression Model. Hepatology (1987) 7(6):1346–58. doi: 10.1002/hep.1840070628 3679094

[34] Moher D Liberati A Tetzlaff J Altman DG Group P . Preferred Reporting Items for Systematic Reviews and Meta-Analyses: The PRISMA Statement. PloS Med (2009) 6(7):e1000097. doi: 10.1371/journal.pmed.1000097 19621072 PMC2707599

[35] Salameh JP Bossuyt PM McGrath TA Thombs BD Hyde CJ Macaskill P . Preferred Reporting Items for Systematic Review and Meta-Analysis of Diagnostic Test Accuracy Studies (PRISMA-DTA): Explanation, Elaboration, and Checklist. BMJ (Clinical Res ed) (2020) 370:m2632. doi: 10.1136/bmj.m2632 32816740

[36] Whiting PF Rutjes AW Westwood ME Mallett S Deeks JJ Reitsma JB . QUADAS-2: A Revised Tool for the Quality Assessment of Diagnostic Accuracy Studies. Ann Internal Med (2011) 155(8):529–36. doi: 10.7326/0003-4819-155-8-201110180-00009 22007046

[37] Hayden JA van der Windt DA Cartwright JL Cote P Bombardier C . Assessing Bias in Studies of Prognostic Factors. Ann Internal Med (2013) 158(4):280–6. doi: 10.7326/0003-4819-158-4-201302190-00009 23420236

[38] Higgins JPT GSe . Cochrane Handbook for Systematic Reviews of Interventions Version 5.1.0 [Updated March 2011]. The Cochrane Collaboration (2011). Available at: http://handbook.cochrane.org.

[39] DerSimonian R Laird N . Meta-Analysis in Clinical Trials. Control Clin Trials (1986) 7(3):177–88. doi: 10.1016/0197-2456(86)90046-2 3802833

[40] Henry L Fabre C Guiraud I Bastide S Fabbro-Peray P Martinez J . Clinical Use of P-Proteasome in Discriminating Metastatic Melanoma Patients: Comparative Study With LDH, MIA and S100B Protein. Int J Cancer (2013) 133(1):142–8. doi: 10.1002/ijc.27991 23238767

[41] Diaz-Lagares A Alegre E Arroyo A Gonzalez-Cao M Zudaire ME Viteri S . Evaluation of Multiple Serum Markers in Advanced Melanoma. Tumour Biol (2011) 32(6):1155–61. doi: 10.1007/s13277-011-0218-x 21858537

[42] Garbe C Leiter U Ellwanger U Blaheta HJ Meier F Rassner G . Diagnostic Value and Prognostic Significance of Protein S-100beta, Melanoma-Inhibitory Activity, and Tyrosinase/MART-1 Reverse Transcription-Polymerase Chain Reaction in the Follow-Up of High-Risk Melanoma Patients. Cancer (2003) 97(7):1737–45. doi: 10.1002/cncr.11250 12655531

[43] Garnier JP Letellier S Cassinat B Lebbe C Kerob D Baccard M . Clinical Value of Combined Determination of Plasma L-DOPA/tyrosine Ratio, S100B, MIA and LDH in Melanoma. Eur J Cancer (Oxford Engl 1990) (2007) 43(4):816–21. doi: 10.1016/j.ejca.2006.11.022 17276671

[44] Mohammed MQ Abraha HD Sherwood RA MacRae K Retsas S . Serum S100beta Protein as a Marker of Disease Activity in Patients With Malignant Melanoma. Med Oncol (Northwood London England) (2001) 18(2):109–20. doi: 10.1385/mo:18:2:109 11778756

[45] Maier T Laubender RP Sturm RA Klingenstein A Korting HC Ruzicka T . Osteopontin Expression in Plasma of Melanoma Patients and in Melanocytic Tumours. J Eur Acad Dermatol Venereology JEADV (2012) 26(9):1084–91. doi: 10.1111/j.1468-3083.2011.04210.x 21838826

[46] Missotten GS Korse CM van Dehn C Linders TC Keunen JE Jager MJ . S-100B Protein and Melanoma Inhibitory Activity Protein in Uveal Melanoma Screening. A Comparison With Liver Function Tests. Tumour Biol (2007) 28(2):63–9. doi: 10.1159/000099151 17264538

[47] Weide B Elsasser M Buttner P Pflugfelder A Leiter U Eigentler TK . Serum Markers Lactate Dehydrogenase and S100B Predict Independently Disease Outcome in Melanoma Patients With Distant Metastasis. Br J Cancer (2012) 107(3):422–8. doi: 10.1038/bjc.2012.306 PMC3405231 22782342

[48] Weide B Richter S Buttner P Leiter U Forschner A Bauer J . Serum S100B, Lactate Dehydrogenase and Brain Metastasis Are Prognostic Factors in Patients With Distant Melanoma Metastasis and Systemic Therapy. PloS One (2013) 8(11):e81624. doi: 10.1371/journal.pone.0081624 24312329 PMC3842933

[49] Weide B Schafer T Martens A Kuzkina A Uder L Noor S . High GDF-15 Serum Levels Independently Correlate With Poorer Overall Survival of Patients With Tumor-Free Stage III and Unresectable Stage IV Melanoma. J Invest Dermatol (2016) 136(12):2444–52. doi: 10.1016/j.jid.2016.07.016 27705749

[50] Amaral T Kiecker F Schaefer S Stege H Kaehler K Terheyden P . Combined Immunotherapy With Nivolumab and Ipilimumab With and Without Local Therapy in Patients With Melanoma Brain Metastasis: A DeCOG* Study in 380 Patients. J Immunother Cancer (2020) 8(1):e000333. doi: 10.1136/jitc-2019-000333 32221017 PMC7206917

[51] Wagner NB Forschner A Leiter U Garbe C Eigentler TK . S100B and LDH as Early Prognostic Markers for Response and Overall Survival in Melanoma Patients Treated With Anti-PD-1 or Combined Anti-PD-1 Plus Anti-CTLA-4 Antibodies. Br J Cancer (2018) 119(3):339–46. doi: 10.1038/s41416-018-0167-x PMC6070917 29950611

[52] Amaral T Schulze M Sinnberg T Nieser M Martus P Battke F . Are Pathogenic Germline Variants in Metastatic Melanoma Associated With Resistance to Combined Immunotherapy? Cancers (2020) 12(5):1101. doi: 10.3390/cancers12051101 PMC7281129 32354124

[53] Damuzzo V Solito S Pinton L Carrozzo E Valpione S Pigozzo J . Clinical Implication of Tumor-Associated and Immunological Parameters in Melanoma Patients Treated With Ipilimumab. Oncoimmunology (2016) 5(12):e1249559. doi: 10.1080/2162402x.2016.1249559 28123888 PMC5215225

[54] Eigentler TK Figl A Krex D Mohr P Mauch C Rass K . Number of Metastases, Serum Lactate Dehydrogenase Level, and Type of Treatment Are Prognostic Factors in Patients With Brain Metastases of Malignant Melanoma. Cancer (2011) 117(8):1697–703. doi: 10.1002/cncr.25631 21472716

[55] Wevers KP Kruijff S Speijers MJ Bastiaannet E Muller Kobold AC . Hoekstra HJ. S-100B: A Stronger Prognostic Biomarker Than LDH in Stage IIIB-C Melanoma. Ann Surg Oncol (2013) 20(8):2772–9. doi: 10.1245/s10434-013-2949-y 23512078

[56] Schmidt H Sorensen BS Fode K Nexo E von der Maase H . Tyrosinase Messenger RNA in Peripheral Blood Is Related to Poor Survival in Patients With Metastatic Melanoma Following Interleukin-2-Based Immunotherapy. Melanoma Res (2005) 15(5):409–16. doi: 10.1097/00008390-200510000-00009 16179868

[57] Sorci G Riuzzi F Arcuri C Tubaro C Bianchi R Giambanco I . S100B Protein in Tissue Development, Repair and Regeneration. World J Biol Chem (2013) 4(1):1–12. doi: 10.4331/wjbc.v4.i1.1 23580916 PMC3622753

[58] Olaoba OT Kadasah S Vetter SW Leclerc E . RAGE Signaling in Melanoma Tumors. Int J Mol Sci (2020) 21(23):8989. doi: 10.3390/ijms21238989 PMC7730603 33256110

[59] Leclerc E Heizmann CW . The Importance of Ca2+/Zn2+ Signaling S100 Proteins and RAGE in Translational Medicine. Front Biosci (Schol Ed) (2011) 3:1232–62. doi: 10.2741/223 21622268

[60] Perez-Tomas R Perez-Guillen I . Lactate in the Tumor Microenvironment: An Essential Molecule in Cancer Progression and Treatment. Cancers (2020) 12(11):3244. doi: 10.3390/cancers12113244 PMC7693872 33153193

[61] Ho J de Moura MB Lin Y Vincent G Thorne S Duncan LM . Importance of Glycolysis and Oxidative Phosphorylation in Advanced Melanoma. Mol Cancer (2012) 11:76. doi: 10.1186/1476-4598-11-76 23043612 PMC3537610

[62] Abraha HD Fuller LC Du Vivier AW Higgins EM Sherwood RA . Serum S-100 Protein: A Potentially Useful Prognostic Marker in Cutaneous Melanoma. Br J Dermatol (1997) 137(3):381–5. doi: 10.1111/j.1365-2133.1997.tb03742.x 9349333

[63] Egberts F Momkvist A Egberts JH Kaehler KC Hauschild A . Serum S100B and LDH Are Not Useful in Predicting the Sentinel Node Status in Melanoma Patients. Anticancer Res (2010) 30(5):1799–805. 20592382

[64] Stark MS Klein K Weide B Haydu LE Pflugfelder A Tang YH . The Prognostic and Predictive Value of Melanoma-Related MicroRNAs Using Tissue and Serum: A MicroRNA Expression Analysis. EBioMedicine (2015) 2(7):671–80. doi: 10.1016/j.ebiom.2015.05.011 PMC4534690 26288839

[65] Slostad JA Liu MC Allred JB Erickson LA Rumilla KM Block MS . BRAF V600 Mutation Detection in Plasma Cell-Free DNA: NCCTG N0879 (Alliance). Mayo Clin Proc Innov Qual Outcomes (2021) 5(6):1012–20. doi: 10.1016/j.mayocpiqo.2021.05.003 PMC8526905 34703985

[66] Moons KGM Wolff RF Riley RD Whiting PF Westwood M Collins GS . PROBAST: A Tool to Assess Risk of Bias and Applicability of Prediction Model Studies: Explanation and Elaboration. Ann Internal Med (2019) 170(1):W1–33. doi: 10.7326/M18-1377 30596876

[67] Kluger HM Hoyt K Bacchiocchi A Mayer T Kirsch J Kluger Y . Plasma Markers for Identifying Patients With Metastatic Melanoma. Clin Cancer Res (2011) 17(8):2417–25. doi: 10.1158/1078-0432.CCR-10-2402 PMC3415234 21487066

[68] Frauchiger AL Dummer R Mangana J . Serum S100B Levels in Melanoma. Methods Mol Biol (2019) 1929:691–700. doi: 10.1007/978-1-4939-9030-6_43 30710305

[69] Felix J Cassinat B Porcher R Schlageter MH Maubec E Pages C . Relevance of Serum Biomarkers Associated With Melanoma During Follow-Up of Anti-CTLA-4 Immunotherapy. Int Immunopharmacol (2016) 40:466–73. doi: 10.1016/j.intimp.2016.09.030 27728898

[70] Sanmamed MF Fernandez-Landazuri S Rodriguez C Lozano MD Echeveste JI Perez Gracia JL . Relevance of MIA and S100 Serum Tumor Markers to Monitor BRAF Inhibitor Therapy in Metastatic Melanoma Patients. Clin Chim Acta (2014) 429:168–74. doi: 10.1016/j.cca.2013.11.034 24333389

[71] Yue C Jiang Y Li P Wang Y Xue J Li N . Dynamic Change of PD-L1 Expression on Circulating Tumor Cells in Advanced Solid Tumor Patients Undergoing PD-1 Blockade Therapy. Oncoimmunology (2018) 7(7):e1438111. doi: 10.1080/2162402x.2018.1438111 29900038 PMC5993493

[72] Abusaif S Jradi Z Held L Pflugfelder A Weide B Meier F . S100B and Lactate Dehydrogenase as Response and Progression Markers During Treatment With Vemurafenib in Patients With Advanced Melanoma. Melanoma Res (2013) 23(5):396–401. doi: 10.1097/CMR.0b013e3283650741 23907232

[73] Gassenmaier M Lenders MM Forschner A Leiter U Weide B Garbe C . Serum S100B and LDH at Baseline and During Therapy Predict the Outcome of Metastatic Melanoma Patients Treated With BRAF Inhibitors. Target Oncol (2021) 16(2):197–205. doi: 10.1007/s11523-021-00792-8 33555543 PMC7935737

